# Perceived benefits of digital health and social services among older adults: A population-based cross-sectional survey

**DOI:** 10.1177/20552076231173559

**Published:** 2023-06-04

**Authors:** Emma Kainiemi, Petra Saukkonen, Lotta Virtanen, Tuulikki Vehko, Maiju Kyytsönen, Mari Aaltonen, Tarja Heponiemi

**Affiliations:** 3837 Finnish Institute for Health and Welfare (THL), Helsinki, Finland

**Keywords:** Older adults, digital health and social care services, benefits from digital services, surveys and questionnaires

## Abstract

**Objective:**

The aim of this study was to describe the benefits of digital health and social services perceived by older adults and to examine factors associated with perceiving these benefits. Several factors related to (a) sociodemographic characteristics, (b) area of residence, (c) physical, cognitive, psychological, and social functioning, and (d) Internet use, were examined.

**Methods:**

The present sample included 8019 respondents aged between 75 and 99 years. The inverse probability weighting method was used to correct for bias. Linear regression analyses were used to examine the associations.

**Results:**

The ease of use of the services regardless of the time and location was perceived as the most beneficial. Convenient distance to local health or social services (parameter estimate  =  0.15 [0.08–0.23]), good functional ability (PE  =  0.08 [0.01–0.14]), good vision (PE  =  0.15 [0.04–0.25]), ability to learn (PE  =  0.05 [0.01–0.10]) and living with someone (PE  =  0.08 [95% CI 0.04–0.13]) were associated with perceiving more benefits. In addition, access to the Internet (PE  =  0.12 [0.06–0.19]) and independent use of the Internet (PE  =  0.23 [0.17–0.29]) were associated with perceiving more benefits.

**Conclusions:**

Older adults who are healthier, have a social relationship in their everyday life or have easier access to traditional services seem to perceive more benefits from digital health and social services. Digital services should be developed to correspond with special needs caused by disadvantages in health and the social environment. To facilitate the use of digital health and social services, more efforts should be made to enhance older adults’ perceptions of their benefits.

## Introduction

Societies worldwide are aging as life expectancy is lengthening.^1^ With the aging of the population the number of older adults living with disabilities and long-term illnesses will also increase.^2,3^ This increases the populations’ health care demands^4,5^ and reliance on social services.^6^ Simultaneously older adults face problems accessing health and social services as provision seems to become increasingly scarce^7,8^ and deterioration in mobility limits their abilities to leave their homes to receive services.^9^

The use of digital services provides older adults with an opportunity to compensate for potential losses in their mobility,^9,10^ improves access to health and social services and supports in the management of one's own health and wellbeing^5,11,12^ Moreover, the use of digital health and social services may foster active aging and help older adults to manage their health and remain independent longer, ultimately promoting better wellbeing and quality of life.^7,12,13^ From the service providers’ perspective the use of digital services has the potential to improve the allocation of scarce resources and the efficiency and cost-effectiveness of the services.^7,11,14^

The dissemination of technology and digital services is increasing^15^ and the coronavirus disease 2019 (COVID-19) pandemic even accelerated the provision and use of digital services as alternatives to in-person visits.^16^ However, the increased provision of digital services has the potential to exacerbate digital exclusion especially among the oldest of the older adults,^17^ enforcing the already existing inequalities in society and in health and wellbeing.^18–20^ Although older adults are increasingly using technology, previous literature indicates that they typically have lower access to and usage of digital technologies^18,21–23^ and are less likely to adopt^22,24^ and learn^25^ current technologies required to access the Internet and digital services compared to younger adults.

Perceptions of the benefits of digital technology, the Internet and digital services are strongly associated with their adoption and use.^5,7,15,26,27^ Older adults have been shown to have less interest in digital services^28^ and perceive fewer benefits^12,18,29^ compared to younger generations. Sociodemographic factors such as male gender^12^ and higher education^26,29^ have been reported to be associated with perceiving more benefits from the use of the Internet and digital services. In addition, potential associations between health status and perceived benefits of digital services have been identified^18^ and declines in physical and cognitive functioning have been reported to be associated with non-use of digital technology and the Internet.^12,30,31^ People living alone have previously perceived fewer benefits of the use of digital services and the Internet^29^ and concerns have been raised regarding the risk of digital exclusion among socially isolated individuals.^18^ Moreover, access to technology^25,27^ and trust in the security of digital services are key in perceiving benefits from these services.^18^

However, previous studies mainly concern the factors associated with the use and perceptions of the benefits of the Internet and digital services in general and adults of all ages have been included as participants. Moreover, most previous studies have included only participants who use the Internet and little research has been conducted using a nationally representative sample of older adults. Given the rapid aging of the population and the increased provision of digital services, it is essential to understand how older adults perceive the benefits of digital health and social services, as their perceptions are strongly associated with the use.^18^

This study aims to evaluate the benefits perceived by older adults of digital health and social services and to examine which factors are associated with these perceived benefits. Several factors related to (a) sociodemographic characteristics, (b) area of residence, (c) physical, cognitive, psychological, and social functioning, and (d) the use of the Internet, were examined. The findings of this study might offer a better understanding of the perceived benefits of digital health and social services and the associated factors. This information could be beneficial in developing future digital interventions to improve the health and wellbeing and quality of life of older adults as well as to increase service utilisation and reduce disparities.

Finland provides a fruitful context for conducting the study as it is one of the fastest-aging societies in Europe^32^ and among leading countries in digitalisation.^33^ In addition, the number of chronic conditions is above the average of the European Union^8^ and the uneven geographic distribution of health and social care resources has been argued to reinforce disparities in access to services.^8,34^

## Methods

### Sample

This study was conducted as a part of the FinSote 2020 National survey of health, wellbeing, and service use in Finland.^35^ The national survey consisted of three questionnaires that were modified for different age categories and included a questionnaire for respondents aged 75 years or more. The questionnaire was sent using stratified sampling to 17,600 Finnish residents who had reached the age of 75 years. A possibility to respond in Finnish, Swedish, Russian, or English, either in digital or paper form, were offered. During the data collection, participants who had not responded were approached by mail up to four times. The data were collected between September 2020 and February 2021 during the second wave of the COVID-19 pandemic.

Altogether 9919 Finnish residents who had reached the age of 75 years (60.3% female, mean age 81.45 years, SE 0.06) responded to the questionnaire (response rate 52.3%). The majority of the respondents (94.7%) participated by answering the questionnaire in paper format. In total, 8019 respondents had responded to the items concerning the perceptions of the benefits of digital health and social services and were included in the analyses. The inverse probability weighting (IPW) method^36^ was used for the received responses to improve the accuracy of the results and remove most of the non-response bias.^37^ The weights were estimated using sociodemographic register-based variables including the respondents’ age, sex, marital status, area of residence, and native language, received from the National Population Register.

### Measurements

#### Dependent variable

*The perceived benefits of digital health and social services* were measured with six statements which were developed based on the Finnish National Strategy for Electronic Health and Welfare 2020.^38^ The respondents were asked to select how they felt about the following statements concerning the benefits of digital health and social services: They (1) help me to assess the need for services, (2) support me in finding and choosing the most suitable service, (3) make it easier for me to use services regardless of where I am and when, (4) make it easier for me to collaborate with professionals, (5) help me to take an active role in looking after my own health and welfare, and (6) help me to take care of the health, welfare and functional capacity of family or friends. A 5-point Likert scale was used to answer the statements (1  =  completely disagree to 5  =  completely agree). A mean variable ranging from one to five was calculated for each respondent to represent the level of how beneficial they perceived digital health and social services. The Cronbach's alpha value for the statements was 0.94, representing excellent internal consistency of the items.^39^

#### Independent variables

Independent variables included characteristics concerning respondents’ (a) sociodemographic background, (b) area of residence, (c) physical, cognitive, psychological, and social functioning, and (d) the use of the Internet. The used independent variables are described more in detail in Supplemental Material.

The respondents’ sociodemographic characteristics included age, sex, and educational level. These were used only as adjustments, as these factors have already been extensively studied.

The area of residence included the degree of urbanisation, determined based on the municipal classification, and distance to local services. The distance to local services was assessed by asking the respondents separate questions about whether difficult access to the place of service had interfered with receiving treatment and social services in the past 12 months.

Factors related to respondents’ physical, cognitive, psychological, and social functioning included functional disability, impaired vision, the ability to learn new things, psychological distress, and living alone. Widely used instruments, Global Activity Limitation Indicator^40^ and Mental Health Inventory^41^ were used to measure the functional disability and psychological distress, respectively. Both instruments have shown good validity and reliability.^42,43^ Access to the Internet, independent use of the Internet, and information security concerns were included as factors related to the use of the Internet.

### Statistical analysis

In all statistical analyses, the IPW method^36^ was applied to correct bias resulting from differential sampling probabilities and missing data. Complex Samples Multiple Linear Regression analyses were used to examine the adjusted associations of respondent characteristics with the perceived benefits of digital health and social services. First, separate analyses (Model 0) were conducted to examine the association of each independent variable with the dependent variable, adjusted for age, sex, and educational level. Second, a fully adjusted multivariable model (Model 1) was formed to measure the joint effects of all the independent variables, adjusted for age, sex, and educational level.

The appropriateness of the linear model was examined for a linear relationship, non-collinearity, normal distribution, homoscedasticity, and non-correlation of residuals. Statistical methods suitable for weighted data were used and SPSS 27 was applied for the analyses. Due to nonresponse in some items, the number of observations varies in the analyses.

## Results

### Characteristics

The characteristics of the respondents are presented in Table 1. The mean age of the respondents was 81.1 years. Over half of the respondents were females and lived in urban municipalities. Most respondents reported having convenient distance to their care provider (87.1%). A minority of the respondents had a severe functional disability (14.8%) or severely impaired vision (6.2%). Approximately two-thirds of the respondents (61.5%) had access to the Internet, and almost half (48.3%) used the Internet independently.3

**Table 1.** The characteristics of the respondents (IPW weighted) *n*  =  8019^a^.

Characteristics			Value
*Sociodemographic characteristics*			
	Age *mean* (SE) (*n* = 8019)		81.1 (.07)
	Sex *n* (%) (*n* = 8019)		
		Male	3553 (41.4)
		Female	4466 (58.6)
	Educational level *n* (%) (*n* = 7491)		
		Low	2560 (36.2)
		Median	2744 (36.8)
		High	2187 (27.0)
*Variables related to area of residence*			
	Degree of urbanisation *n* (%) (*n* = 8019)		
		Non-urban	3641 (43.3)
		Urban	4378 (56.7)
	Distance to local services *n* (%) (*n* = 7082)		
		Convenient	6218 (87.1)
		Inconvenient	864 (12.9)
*Physical, cognitive, psychological, and social functioning*			
	Functional disability *n* (%) (*n* = 7695)		
		Mild or non	6691 (85.2)
		Severe	1004 (14.8)
	Severely impaired vision *n* (%) (*n* = 7854)		
		No	7431 (93.8)
		Yes	423 (6.2)
	Ability to learn new things *n* (%) (*n* = 7813)		
		Good	2844 (33.8)
		Poor or average	4969 (66.2)
	Psychological distress *n* (%) (*n* = 7855)		
		No	7095 (89.7)
		Yes	760 (10.3)
	Living alone *n* (%) (*n* = 7664)		
		No	4454 (52.8)
		Yes	3210 (47.2)
*The use of the internet*			
	Access to the internet *n* (%) (*n* = 7788)		
		No	2729 (38.5)
		Yes	5059 (61.5)
	Independent use of the internet *n* (%) (*n* = 7972)		
		No	3874 (51.7)
		Yes	4098 (48.3)
	Information security concerns *n* (%) (*n* = 7033)		
		No	3935 (55.7)
		Yes	3098 (44.3)

^a^ Inverse Probability Weighting (IPW) corrected percentiles, *n*-size uncorrected.

### Perceived benefits

**Figure 1.**
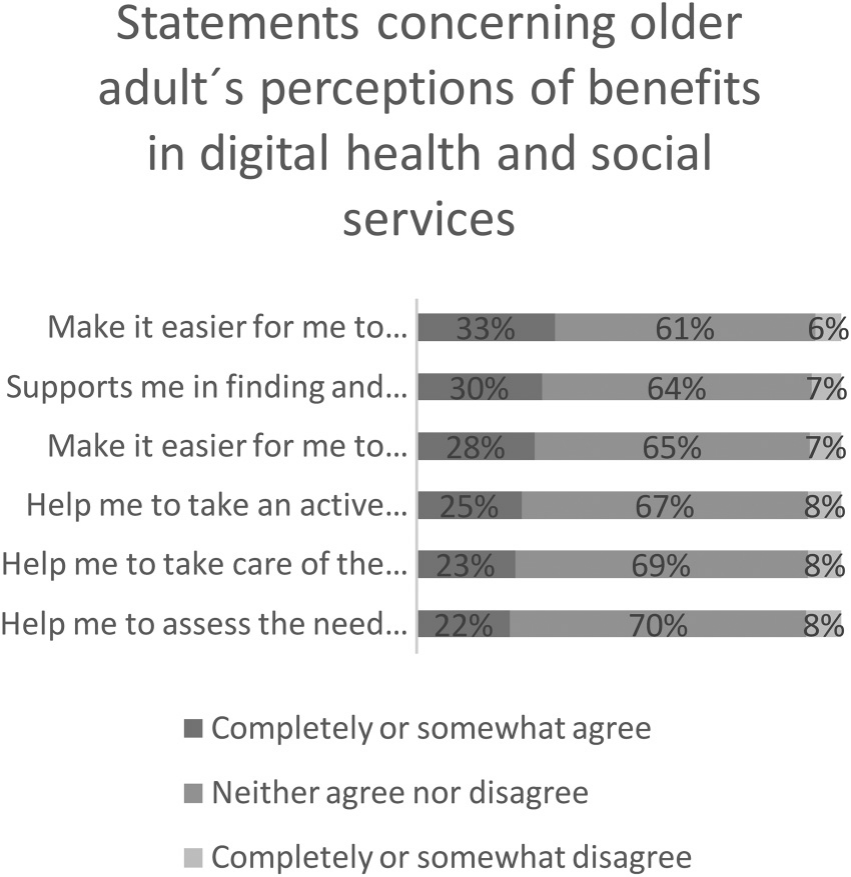
Perceived benefits of digital health and social services by older adults (*n*  =  8019, weighted percentiles).

The distribution of the perceived benefits of digital health and social services experienced by older adults are described in detail in Figure 1. The respondents mostly had neutral perceptions of the benefits of digital health and social services (mean 3.23, SE .01), as most older adults did not agree or disagree with the statements describing the benefits.

One-third (33%) of older adults perceived that digital health and social services eased the use of the services regardless of the time and location. Slightly under one in three perceived that digital health and social services support them in finding and choosing the most suitable service (30%) and made collaboration with professionals easier (28%). The respondents found the following statements less beneficial: digital health and social services help in taking an active role in looking after one's own health and welfare (25%), help to take care of the health, welfare and functional capacity of family or friends (23%) and help to assess the need for services (22%).

Table 2 shows the results of multivariable linear regression Model 1 for perceived benefits of digital health and social services. Model 1 explained 11% of the variance (*R*²) in perceiving benefits of digital services. In Model 1, living alone, the distance to local health or social care services, physical and cognitive functioning, and access to the Internet and ability to use it independently remained statistically significant variables associated with perceiving benefits of digital health and social services. Older adults who experienced that their local health or social services were convenient to reach perceived more benefits from digital health and social services compared to their counterparts with inconvenient proximity. Older adults with good functional ability or vision and with a good ability to learn, perceived digital health and social services to be more beneficial than their counterparts with severely impaired functional ability or vision and with a poor ability to learn. Older adults who lived together with someone perceived digital health and social services as more beneficial than those who lived alone. Older adults who had access to and used the Internet independently perceived more benefits from digital health and social services than those without access and independent use of the Internet.

**Table 2.** The results of linear regression analyses for perceived benefits of digital health and social services (*n*  =  5329)^a^.

Characteristics			Model 0^b^			Model 1^c^		
			Parameter estimate^d^	95% CI	*P*-value	Parameter estimate^d^	95% CI	*P*-value
*Sociodemographic characteristics*								
	Age		−0.02	−0.02–−0.02	<0.001	0.00	−0.01–0.01	0.632
	Sex							
		Female	Reference			Reference		
		Male	0.11	0.08–0.15	<0.001	0.07	0.02–0.11	0.004
	Educational level				<0.001			0.048
		Low	Reference			Reference		
		Median	0.14	0.10–0.18		0.06	0.01–0.11	
		High	0.26	0.20–0.30		0.06	0.01–0.12	
*Variables related to area of residence*								
	Degree of urbanisation							
		Non-urban	Reference			Reference		
		Urban	0.04	0.01–0.08	0.017	0.02	−0.03–0.06	0.463
	Distance to local services							
		Inconvenient	Reference			Reference		
		Convenient	0.17	0.10–0.24	<0.001	0.15	0.08–0.23	<0.001
*Physical, cognitive, psychological, and social functioning*								
	Functional disability							
		Severe	Reference			Reference		
		Mild or non	0.17	0.11–0.23	<0.001	0.08	0.01–0.14	0.024
	Severely impaired vision							
		Yes	Reference			Reference		
		No	0.25	0.15–0.34	<0.001	0.15	0.04–0.25	0.005
	Ability to learn new things							
		Poor or average	Reference			Reference		
		Good	0.13	0.09–0.17	<0.001	0.05	0.01–0.10	0.022
	Psychological distress							
		No	Reference			Reference		
		Yes	0.07	0.00–0.14	0.046	0.03	−0.05–0.12	0.446
	Living alone							
		Yes	Reference			Reference		
		No	0.07	0.03–0.11	<0.001	0.08	0.04–0.13	<0.001
*The use of the Internet*								
	Access to the Internet							
		No	Reference			Reference		
		Yes	0.32	0.27–0.36	<0.001	0.12	0.06–0.19	<0.001
	Independent use of the internet							
		No	Reference					
		Yes	0.35	0.31–0.39	<0.001	0.23	0.17–0.29	<0.001
	Information security concerns							
		No	Reference			Reference		
		Yes	0.05	0.01–0.09	0.010	0.02	−0.02–0.06	0.354

^a^ Inverse Probability Weighting (IPW) corrected.

^b^ Model 0 included the main effect of each variable.

^c^ Model 1 included all the examined variables.

^d^ Parameter estimate represents the amount of change in dependent variable if independent variable changes by one unit.

95% CI: 95% confidence interval of parameter estimate.

## Discussion

The study aimed to evaluate the benefits perceived by older adults of digital health and social services and to examine which factors are associated with these perceived benefits. Older adults who participated in this nationally representative survey study had predominantly neutral perceptions of the benefits of digital health and social services. Facilitation of the use of services regardless of time and location was perceived as the greatest benefit of digital health and social services. Improved access and convenience have also previously been found as perceived benefits of digital services,^44^ for example, because digital services reduce the need for physical queuing or travelling to reach the traditional place of service provider.^14,45^ Digital technology has been perceived to be a practical, inexpensive and good alternative to traditional services among older adults.^44^ The overall neutral perceptions could be anticipated to reflect the fact that digital services in the Finnish field of social care have not been as widely used as in health care,^45^ and many may not have specific expectations or perceptions of them. However, the most used digital health and social service, the national patient portal My Kanta Pages, has gained satisfaction among older adults.^46^ The citizens’ might be anticipated to have capabilities for wider adoption of digital social services because many are already familiar with digital health services.^45^

Respondents who had a convenient distance to their local health or social services perceived more benefits from digital services than their counterparts, even after adjusting for the degree of urbanisation. The result is unexpected, as digital health and social services have aimed to improve access to services, regardless of the distance to the local services.^11,45,47^ Previous studies have observed that urban populations seem to have fewer health concerns compared to rural areas.^48,49^ This could partly explain the results as high and complicated service needs are more difficult to fulfil with digital services. On the other hand, the finding is an alert to the question of whether older adults in rural environments receive enough information about digital services. The fact that older adults in the present study reported the facilitation of the use of services regardless of time and location as the most beneficial aspect of digital health and social services is encouraging and particularly important in sparsely populated countries such as Finland.

Learning new things requires high cognitive involvement^9^ and capabilities for processing and reasoning, which generally decline among the aging.^15^ Cognitive disabilities are associated with poor digital competence and the non-use of digital technology, the Internet, and digital services.^47,50–54^ In the present study, older adults with a good ability to learn new things perceived digital health and social services to be more beneficial than their counterparts with a poor ability to learn new things. This is similar to previous research indicating that normal cognitive functioning is associated with perceiving digital health services to be more beneficial.^11^ The result is expected but worrying because with the aging of the population, for example, every year thousands of people are diagnosed with dementia and have cognitive problems.^55^ Digital health and social services do not yet seem to serve the needs of these people. However, the use of digital technology could stimulate older adults’ cognitive abilities and functioning^23^ by offering them an intellectual learning activity.^51,56^ This would require simplified language in digital services and more pronounced and planned support for the use of digital services.

In the present study, older adults without severe functional disabilities perceived digital health and social services as more beneficial than their severely disabled counterparts. Experiencing physical limitations seems to have a significant impact on older adults’ ability to use technology.^57,58^ Previous research has discovered an association between functional limitations and non-use of the Internet among older adults,^25,50^ and computer users have been reported to be significantly healthier than nonusers.^59^ Moreover, poor health has predicted the low use of digital services among older adults,^17^ and concerns have been raised about whether older adults with limited functioning benefit from digital services.^54^ The use of technology could improve the quality of life of older adults by increasing their ability to perform tasks and remain independent for longer^60^ and digital health services have the potential to improve their health and wellbeing.^61,62^ However, to accomplish these benefits, acceptance and use of digital services by older adults is pivotal.^50^

Limitations in vision often occur with aging^63^ and pose challenges that discourage older adults from using the Internet.^64^ It has previously been noted that older adults with vision impairments are less likely to use the Internet^17^ and technology than older adults without impairments in their vision.^50^ However, Internet use could enable older adults with vision impairments to access a wealth of information and peer support that would otherwise be inaccessible.^31^ In the present study, older adults with good vision perceived digital health and social services as more beneficial than their counterparts with severely impaired vision. This might indicate that older adults with vision impairments are unaware of all the possibilities that using the Internet and digital services could offer, that they lack the assistive technology they need or that the services available are designed only for people with good vision. Health and social professionals have a key role in referring visually impaired older adults to suitable digital services and in providing guidance on where to apply for the necessary assistive technology. In Finland, assistive technology for visually impaired persons is provided by central hospitals or the Social Insurance Institution (Kela).^65^

More and more older people are living alone as the population ages.^66^ Previous literature has highlighted the importance of social participation and the presence of a partner for using the Internet^18,29,47,59^ and older adults who live alone have been reported to benefit less from the use of the Internet.^29^ According to the results of the present study, older adults who lived together with someone perceived digital health and social services to be more beneficial than those who lived alone. It has been previously reported that living with someone increases the likelihood of being encouraged and supported to use the Internet.^47,67^ Further, it has been presumed that the range of available digital devices and services is wider for older adults who gain support from a person that is close to them.^44^ To increase the positive perceptions and the use of digital health and social services, it is necessary to ensure that older adults who live alone have equal possibilities to access technology and digital services as those not living alone.

According to the results of the present study, older adults who had access to the Internet and used the Internet independently perceived more benefits from digital health and social services than their counterparts without access and independent use. This is congruent with previous research indicating that Internet access and having the necessary devices for use were strongly associated with more positive perceptions and acceptance of digital services in the field of health care.^11^ Further, the use of digital health services and perceived benefits have been recognised to be largely dependent on prior use.^68^ Older adults who are not engaged with technology and the Internet might not have realised the benefits they could achieve by using them. More efforts are needed to increase their awareness of different digital health and social services and the benefits they might bring.^11,14^

### Limitations

There are some limitations to the present study that need to be considered when interpreting the results. The findings are based on self-reported data, offering a possibility for recall bias. This could also lead to problems associated with common method variance and the inflation of the strength of relationships. Although multiple factors were adjusted in the analyses, the possibility of residual confounding remains, and it is possible that some other variables which we did not examine might have affected the results. Moreover, because cross-sectional data were used, causal inferences cannot be drawn from the results. Some of the selected variables are hard to explicitly measure and quantify because of their subjective nature, such as the dependent variable about the perceived benefits of digital health and social services. Furthermore, the psychometric properties of the dependent measure have not been tested previously. However, the statements are based on the Finnish eHealth and eSocial Strategy 2020^38^ and describe the benefits of digital services from the perspective of the focus areas included in the strategy.

One limitation of the present study was the central tendency bias of the perceived benefits of digital health and social services, meaning that many respondents selected the neutral “not agree nor disagree” option. It has been previously observed that in Likert-scale questionnaires respondents usually avoid ends of the scale in their answers and wish to be conservative by responding with the middle option.^69^ In addition, the respondents were instructed to answer the dependent variable as follows in our study: “If you cannot assess the digital services, choose neither agree nor disagree”. Thus, we are not aware if a neutral rating was based on a truly neutral opinion or whether the respondent could not assess the digital services, for example, due to the lack of experience or understanding. The perceptions may also be more negative in the target group, as participation in the study required filling out a digital or paper form, which might have excluded the most disadvantaged older adults from the study. In addition, the majority of the older adults who participated in the study did not have severe functional disabilities indicating that the respondents were presumably healthier than the target population.

The unbalance between the amount of research conducted in healthcare and social care made the comparison of our results with previous research healthcare oriented. The research concerning digital social services and especially individuals’ perceptions of the benefits are limited. This has previously been identified and national as well as EU initiatives related to digitalisation of health and social care tend to focus on health-related issues. The digitalisation of social services often takes place in combination with healthcare.^7^

Finland can be considered one of the leading countries in digitalisation^33^ with a well-developed digital infrastructure and near-universal Internet access with high-speed connections. Thus, the results of the present study should be generalised into other environments with caution.

### Implications for the future

Policies are especially needed that support the accessible design of digital services to increase the opportunities for disabled individuals to use the services.^31,50^ Nationwide recommendations^70^ and national legislation^71^ have been set to increase the usability of digital services and equal opportunities for all individuals to access the services. Despite policy implications, many providers of digital health and social services still struggle to meet the recommendations of assessing and reporting the accessibility of their Internet service.^72,73^ More efforts are needed to stimulate the fulfilment of the accessibility requirements. Strategies are needed on how to motivate older adults to engage with assistive technology^74^ and to increase disabled individuals’ awareness of the existing assistive technology and the benefits they could bring.^31^ The role between governmental and non-governmental organisations should be clarified and assistive technology and guidance in their use should be easily available.

Older adults have typically not been considered properly in the design processes of digital services which have mostly been developed by younger generations and according to their digital culture and skills.^75^ Thus, including older adults in the development of digital health and social services could have the potential to ensure that the functions and features of the service meet older adults’ needs and capabilities.^30,45,76^ Services should be easy to use and attention should be paid to the layout and colour schemes, easily understandable language and interfaces that reduce the reliance on perceptual speed and memory.^10,14,64,74,77^ Usability tests could be optimised to focus on accessibility and gather data from representative users among older adults.^78^

In order to increase the positive perceptions of the benefits of digital health and social services, older adults should be provided with opportunities to try unfamiliar technologies and digital services in a supported environment.^77^ Professionals are in a key position in terms of their role promoting the use of digital health and social services and more efforts should be made to increase their possibilities to identify older adults who would especially benefit from the use of such services. If older adults are not aware of the services,^14,76^ their opportunities to benefit from them will remain limited. In addition, the availability of assistance on multiple channels^17^ could enhance the older adults’ use of digital services. Difficulty or unwillingness to ask for technical help either from relatives, friends, or help desks has been reported to hinder their experimentation.^44^

Finally, digital health and social services are a good complement to traditional services^14^ but they should not prevent individuals from choosing to encounter their service provider face-to-face if preferred.^79^ It is crucial to acknowledge that the ability to use digital services is very likely to become more difficult or even impossible at some point during the aging process and thus traditional face-to-face services should be ensured for everyone.^17,54^ It could also be anticipated that individuals might have more positive perceptions of digital health and social services if they feel that they can use them based on their own choice and not because they are obligated to do so.

More research should be targeted on the digitalisation of social services from the point of view of the clients. While some issues and services are similar in the fields of health and social care, a more specific approach on social services would be beneficial for the successful implementation of digital social services.^7^ In addition, research should take into account the multidimensionality of the factors that are associated with the use and perceptions of the benefits of digital health and social services. The possible interactions in independent variables should be acknowledged in further research.

## Conclusions

According to the results of the present population-based cross-sectional study in the COVID-19 era, older adults perceived the ease of use of the services regardless of the time and location as most beneficial in digital health and social services. It seems that more advantaged older adults perceived more benefits in digital health and social services compared to their more disadvantaged counterparts. These findings emphasise the conclusion that digital health and social services are most beneficial to those for whom the use is the easiest and most convenient. Deterioration of health and disadvantages in social environment should be considered in the development of digital health and social services, requiring special sensitivity to individual needs. More efforts should be made to ensure that all individuals have the possibility to try technological devices and digital health and social services in a supportive environment. It is also necessary to highlight that all individuals will age and eventually face challenges in their ability to use digital services if the development of the services does not acknowledge age-related needs more distinctly.

## Supplemental Material

sj-docx-1-dhj-10.1177_20552076231173559 - Supplemental material for Perceived benefits of digital health and social services among older adults: A population-based cross-sectional survey Click here for additional data file. Supplemental material, sj-docx-1-dhj-10.1177_20552076231173559 for Perceived benefits of digital health and social services among older adults: A population-based cross-sectional survey by Emma Kainiemi, Petra Saukkonen, Lotta Virtanen, Tuulikki Vehko, Maiju Kyytsönen, Mari Aaltonen and Tarja Heponiemi in DIGITAL HEALTH

## References

[1] European Observatory on Health Systems and Policies. Ageing and Health: the politics of better policies, https://eurohealthobservatory.who.int/publications/m/ageing-and-health-the-politics-of-better-policies (2021, accessed 13 February 2023). 35951778

[2] Christensen K Doblhammer G Rau R , et al. Ageing populations: the challenges ahead. The Lancet 2009; 374: 1196–1208. 10.1016/S0140-6736(09)61460-4 PMC2810516 19801098

[3] OECD, European Union. Health at a Glance: Europe 2020: State of health in the EU Cycle. OECD Publishing, Paris, 2020. DOI: 10.1787/82129230-en.

[4] European Parliament. Older people in the European Union’s rural areas: issues and challenges: In depth analysis. Directorate General for Parliamentary Research Services. LU: publications office, https://data.europa.eu/doi/10.2861/114962 (2020, accessed 17 May 2022).

[5] Jimison H Gorman P Woods S , et al. Barriers and drivers of health information technology use for the elderly, chronically ill, and underserved. Evid Rep Technol Assess (Full Rep) 2008; 175: 1–58. PMC4781044 19408968

[6] University of Bolton. How does the ageing population affect social care. University of Bolton, https://www.bolton.ac.uk/blogs/how-does-the-ageing-population-affect-social-care/(2022, accessed 14 February 2023).

[7] European Foundation for the Improvement of Living and Working Conditions. Impact of digitalisation on social services. Eurofound. https://www.eurofound.europa.eu/publications/report/2020/impact-of-digitalisation-on-social-services (2020, accessed 14 February 2023).

[8] OECD/European Observatory on Health Systems and Policies. Finland: Country health profile 2021. State of health in the EU. Brussels: OECD Publishing, Paris/European Observatory on Health Systems and Policies, 2021, https://ec.europa.eu/health/system/files/2021-12/2021_chp_fi_english.pdf.

[9] Mcmellon CA Schiffman LG . Cybersenior empowerment: how some older individuals are taking control of their lives. J Appl Gerontol 2002; 21: 157–175.

[10] Hill R Betts LR Gardner SE . Older adults’ experiences and perceptions of digital technology: (Dis)empowerment, wellbeing, and inclusion. Comput Human Behav 2015; 48: 415–423.

[11] Bujnowska-Fedak MM Pirogowicz I . Support for e-health services among elderly primary care patients. Telemed J E Health 2014; 20: 696–704. 24359252 10.1089/tmj.2013.0318 PMC4106384

[12] Czaja SJ Charness N Fisk AD , et al. Factors predicting the use of technology: findings from the center for research and education on aging and technology enhancement (CREATE). Psychol Aging 2006; 21: 333–352. 16768579 10.1037/0882-7974.21.2.333 PMC1524856

[13] Quittschalle J Stein J Luppa M , et al. Internet use in old age: results of a German population-representative survey. J Med Internet Res 2020; 22: e15543. 33226351 10.2196/15543 PMC7685698

[14] Jung M-L Loria K . Acceptance of Swedish e-health services. J Multidiscip Healthc 2010; 3: 55–63. 21289860 10.2147/JMDH.S9159 PMC3024889

[15] Czaja SJ Lee CC . The impact of aging on access to technology. Univ Access Inf Soc 2007; 5: 341–349.

[16] Ting DSW Carin L Dzau V , et al. Digital technology and COVID-19. Nat Med 2020; 26: 459–461. 32284618 10.1038/s41591-020-0824-5 PMC7100489

[17] Heponiemi T Virtanen L Kaihlanen A-M , et al. Use and changes in the use of the internet for obtaining services among older adults during the COVID-19 pandemic: a longitudinal population-based survey study. New Media Soc 2022. Epub ahead of print. DOI: 10.1177/14614448221097000.

[18] Heponiemi T Jormanainen V Leemann L , et al. Digital divide in perceived benefits of online health care and social welfare services: national cross-sectional survey study. J Med Internet Res 2020; 22: e17616. 32673218 10.2196/17616 PMC7381057

[19] Graetz I Gordon N Fung V , et al. The digital divide and patient portals: internet access explained differences in patient portal use for secure messaging by age, race, and income. Med Care 2016; 54: 772–779. 27314262 10.1097/MLR.0000000000000560

[20] Heponiemi T Gluschkoff K Leemann L , et al. Digital inequality in Finland: access, skills and attitudes as social impact mediators. New Media Soc 2021. Epub ahead of print. DOI:10.1177/14614448211023007.

[21] Kumar D Hemmige V Kallen MA , et al. Mobile phones may not bridge the digital divide: a look at mobile phone literacy in an underserved patient population. Cureus 2019; 11: e4104. 31057998 10.7759/cureus.4104 PMC6476614

[22] Niehaves B Plattfaut R . Internet adoption by the elderly: employing IS technology acceptance theories for understanding the age-related digital divide. Eur J Inf Syst 2014; 23: 708–726.

[23] Wu Y-H Lewis M Rigaud A-S . Cognitive function and digital device use in older adults attending a memory clinic. Gerontol Geriatr Med 2019; 5: 2333721419844886. 31080848 10.1177/2333721419844886 PMC6498770

[24] Renaud K Karen Biljon J , et al. Predicting technology acceptance and adoption by the elderly: a qualitative study. 2008. Epub ahead of print. DOI: 10.1145/1456659.1456684.

[25] Lee B Chen Y Hewitt L . Age differences in constraints encountered by seniors in their use of computers and the internet. Comput Human Behav 2011; 27: 1231–1237.

[26] Blank G Lutz C . Benefits and harms from internet use: a differentiated analysis of Great Britain. New Media Soc 2018; 20: 618–640.

[27] Helsper EJ . A corresponding fields model for the links between social and digital exclusion. Commun Theory 2012; 22: 403–426.

[28] Helsper E Reisdorf B . A quantitative examination of explanations for reasons for internet nonuse. Cyberpsychol Behav Soc Netw 2012; 16: 94–99. DOI: 10.1089/cyber.2012.0257. 23249243 10.1089/cyber.2012.0257

[29] van Deursen AJAM Helsper E . The third-level digital divide: who benefits most from being online? 2015: 29–52.

[30] Sakaguchi-Tang DK Bosold AL Choi YK , et al. Patient portal use and experience among older adults: systematic review. JMIR Med Inform 2017; 5: e38. 29038093 10.2196/medinform.8092 PMC5662789

[31] Hollier SE . The disability divide: a study into the impact of computing and internet-related technologies on people who are blind or vision impaired, https://ecommons.cornell.edu/handle/1813/76596 (2007, accessed 3 June 2022).

[32] Pirhonen J Lolich L Tuominen K , et al. “These devices have not been made for older people’s needs” – older adults’ perceptions of digital technologies in Finland and Ireland. Technol Soc 2020; 62: 101287.

[33] European Commission DESI. Shaping Europe’s digital future, https://digital-strategy.ec.europa.eu/en/policies/desi (2021, accessed 20 December 2021).

[34] Keskimäki I Sinervo T Koivisto J , et al. Integrating health and social services in Finland: regional and local initiatives to coordinate care. Public Health Panorama 2018; 04: 679–687.

[35] Finnish Institute for Health and Welfare. National FinSote Survey. Finnish Institute for Health and Welfare (THL), Finland, https://thl.fi/en/web/thlfi-en/research-and-development/research-and-projects/national-finsote-survey (2021, accessed 8 November 2021).

[36] Seaman S White I . Review of inverse probability weighting for dealing with missing data. Stat Methods Med Res 2011; 22: 278–295. DOI: 10.1177/0962280210395740. 21220355 10.1177/0962280210395740

[37] Härkänen T Kaikkonen R Virtala E , et al. Inverse probability weighting and doubly robust methods in correcting the effects of non-response in the reimbursed medication and self-reported turnout estimates in the ATH survey. BMC Public Health 2014; 14: 1150. 25373328 10.1186/1471-2458-14-1150 PMC4246429

[38] Ministry of Social Affairs and Health. Information to support well-being and service renewal. eHealth and eSocial Strategy 2020. https://julkaisut.valtioneuvosto.fi/handle/10024/74459 (2015, accessed 18 January 2022).

[39] Taber KS . The use of Cronbach’s alpha when developing and reporting research instruments in science education. Res Sci Educ 2018; 48: 1273–1296.

[40] Bogaert P Van Oyen H Beluche I , et al. The use of the global activity limitation indicator and healthy life years by member states and the European Commission. Arch Public Health 2018; 76: 30. 29988309 10.1186/s13690-018-0279-z PMC6022353

[41] Berwick DM Murphy JM Goldman PA , et al. Performance of a five-item mental health screening test. Med Care 1991; 29: 169–176. 1994148 10.1097/00005650-199102000-00008

[42] Van Oyen H Bogaert P Yokota RTC , et al. Measuring disability: a systematic review of the validity and reliability of the Global Activity Limitations Indicator (GALI). Arch Public Health 2018; 76: 25. 29881544 10.1186/s13690-018-0270-8 PMC5985596

[43] McHorney CA Ware JE . Construction and validation of an alternate form general mental health scale for the Medical Outcomes Study Short-Form 36-Item Health Survey. Med Care 1995; 33: 15–28. 7823644 10.1097/00005650-199501000-00002

[44] Hänninen R Pajula L Korpela V , et al. Individual and shared digital repertoires – older adults managing digital services. Inform Commun Soc 2021; 26: 568–583.

[45] Kauppila T Kiiski K Lehtonen M . Sähköhelmenkalastus – Sosiaalihuollon sähköisten palvelujen nykytila ja kehittämistarpeet, https://julkaisut.valtioneuvosto.fi/handle/10024/160653 (2018, accessed 17 February 2023).

[46] Kainiemi E Vehko T Kyytsönen M , et al. The factors associated with nonuse of and dissatisfaction with the national patient portal in Finland in the era of COVID-19: population-based cross-sectional survey. JMIR Med Inform 2022; 10: e37500. 35404831 10.2196/37500 PMC9037616

[47] Berner J Rennemark M Jogréus C , et al. Factors influencing internet usage in older adults (65 years and above) living in rural and urban Sweden. Health Inform J 2015; 21: 237–249. 10.1177/1460458214521226 24567416

[48] Jayathilaka R Joachim S Mallikarachchi V , et al. Do chronic illnesses and poverty go hand in hand? PLoS One 2020; 15: e0241232. 33095818 10.1371/journal.pone.0241232 PMC7584216

[49] Saarsalmi P Koskela T Virtala E , et al. Terveyden ja hyvinvoinnin erot maalla ja kaupungissa vuonna 2013 – ATH-tutkimuksen tuloksia uuden kaupunkimaaseutu-luokituksen mukaan. Tutkimuksesta tiiviisti 2014; 30: 1–8. http://urn.fi/URN:ISBN:978-952-302-404-5.

[50] Gell NM Rosenberg DE Demiris G , et al. Patterns of technology use among older adults with and without disabilities. Gerontologist 2015; 55: 412–421. 24379019 10.1093/geront/gnt166 PMC4542705

[51] Choi EY Wisniewski KM Zelinski EM . Information and communication technology use in older adults: a unidirectional or bi-directional association with cognitive function? Comput Human Behav 2021; 121: 106813. 33986562 10.1016/j.chb.2021.106813 PMC8112580

[52] Heo J Chun S Lee S , et al. Internet use and well-being in older adults. Cyberpsychol Behav Soc Netw 2015; 18: 268–272. DOI:10.1089/cyber.2014.0549. 25919967 10.1089/cyber.2014.0549

[53] Heponiemi T Kainiemi E Virtanen L , et al. Performance tests of visual, physical, and cognitive functioning predict Internet use and digital competence among older adults: a longitudinal population-based study. Accepted to J Med Internet Res. 10.2196/42287 PMC10199390 37145836

[54] Saukkonen P Kainiemi E Virtanen L , et al.Non-use of digital services among older adults during the second wave of COVID-19 pandemic in Finland: population-based survey study. In: Gao Q Zhou J (eds) Human Aspects of IT for the Aged Population. Design, Interaction and Technology Acceptance. HCII 2022. Lecture Notes in Computer Science, vol. 13330. Cham: springer.

[55] Finnish Institute for Health and Welfare (THL). Memory disorders. Finnish Institute for Health and Welfare (THL), Finland, https://thl.fi/en/web/chronic-diseases/memory-disorders (2022, accessed 21 September 2022).

[56] Hertzog C Kramer AF Wilson RS , et al. Enrichment effects on adult cognitive development: can the functional capacity of older adults be preserved and enhanced? Psychol Sci Public Interest 2008; 9: 1–65. 26162004 10.1111/j.1539-6053.2009.01034.x

[57] International Telecommunication Union. Ageing in a digital world – from vulnerable to valuable. 2021; 52.

[58] Keränen NS Kangas M Immonen M , et al. Use of information and communication technologies among older people with and without frailty: a population-based survey. J Med Internet Res 2017; 19: e29. 28196791 10.2196/jmir.5507 PMC5331186

[59] Cresci MK Yarandi HN Morrell RW . The digital divide and urban older adults. CIN: Comput Inform Nurs 2010; 28: 88–94. 20182159 10.1097/NCN.0b013e3181cd8184

[60] Erickson J Johnson GM . Internet use and psychological wellness during late adulthood. Can J Aging/La Revue Canadienne du Vieillissement 2011; 30: 197–209. 10.1017/S0714980811000109 24650669

[61] Lim S Kang SM Shin H , et al. Improved glycemic control without hypoglycemia in elderly diabetic patients using the ubiquitous healthcare service, a new medical information system. Diabetes Care 2011; 34: 308–313. 21270188 10.2337/dc10-1447 PMC3024339

[62] Wijsman CA Westendorp RG Verhagen EA , et al. Effects of a web-based intervention on physical activity and metabolism in older adults: randomized controlled trial. J Med Internet Res 2013; 15: e233. 24195965 10.2196/jmir.2843 PMC3841355

[63] National Institute on Aging. Aging and Your Eyes. National Institute on Aging, https://www.nia.nih.gov/health/aging-and-your-eyes (2021, accessed 8 June 2022).

[64] Okonji P Lhussier M Bailey C , et al. Internet use: perceptions and experiences of visually impaired older adults. J Soc Inclus 2015; 6: 121.

[65] Finnish Federation of the Visually Impaired. Tietoa apuvälineistä ja valaistuksesta, https://www.nkl.fi/fi/tietoa-apuvalineista-ja-valaistuksesta (2020, accessed 7 September 2022).

[66] Findlay RA . Interventions to reduce social isolation amongst older people: where is the evidence? Ageing Soc 2003; 23: 647–658.

[67] Friemel TN . The digital divide has grown old: determinants of a digital divide among seniors. New Media Soc 2016; 18: 313–331.

[68] Zhao JY Song B Anand E , et al. Barriers, facilitators, and solutions to optimal patient portal and personal health record use: a systematic review of the literature. AMIA Annu Symp Proc 2018; 2017: 1913–1922. 29854263 PMC5977619

[69] Foddy WH. Constructing questions for interviews and questionnaires: theory and practice in social research. New York, NY, US: Cambridge University Press, 1993.

[70] W3C Web Accessibility Initiative (WAI). WCAG 2 Overview. Web Accessibility Initiative (WAI), https://www.w3.org/WAI/standards-guidelines/wcag/(2022, accessed 13 September 2022).

[71] Finlex. Laki digitaalisten palvelujen tarjoamisesta 306/2019, https://www.finlex.fi/fi/laki/alkup/2019/20190306 (2019, accessed 31 March 2022).

[72] Hospital District of Helsinki and Uusimaa. Accessibility statement for hus.fi. *HUS*, https://www.hus.fi/en/accessibility-statement-husfi (2022, accessed 14 September 2022).

[73] The South Savo social and health care authority. Essoten saavutettavuusseloste. *Essote*, https://www.essote.fi/essoten-saavutettavuusseloste/(2022, accessed 14 September 2022).

[74] Sharit J Moxley JH Boot WR , et al. Effects of extended use of an age-friendly computer system on assessments of computer proficiency, attitudes, and usability by older non-computer users. ACM Trans Access Comput 2019; 12: 9:1–9:28.

[75] Hanson VL . Influencing technology adoption by older adults. Interact Comput 2010; 22: 502–509.

[76] Wildenbos GA Peute L Jaspers M . Facilitators and barriers of electronic health record patient portal adoption by older adults: a literature study. Stud Health Technol Inform 2017; 235: 308–312. 28423804

[77] LeRouge C Van Slyke C Seale D , et al. Baby boomers’ adoption of consumer health technologies: survey on readiness and barriers. J Med Internet Res 2014; 16: e200. 25199475 10.2196/jmir.3049 PMC4180340

[78] World Wide Web Consortium (W3C). Involving Users in Evaluating Web Accessibility. *Web Accessibility Initiative (WAI)*, https://www.w3.org/WAI/test-evaluate/involving-users/(2021, accessed 8 June 2022).

[79] Stanberry B . Telemedicine: barriers and opportunities in the 21st century. J Intern Med 2000; 247: 615–628. 10886483 10.1046/j.1365-2796.2000.00699.x

