# Supplementary material for: Perceived benefits of digital health and social services among older adults: A population-based cross-sectional survey
Source: Digit Health. 2023 Jun 4;9:20552076231173559. doi: 10.1177/20552076231173559 (PMC10259144; doi:10.1177/20552076231173559)
Supplement: sj-docx-1-dhj-10.1177_20552076231173559 - Supplemental material for Perceived benefits of digital health and social services among older adults: A population-based cross-sectional survey [file sj-docx-1-dhj-10.1177_20552076231173559.docx]

INDEPENDENT VARIABLES

| **Variable** | | **Item in questionnaire** | **Response options** | **Coding in the analyses** |
| --- | --- | --- | --- | --- |
| *Sociodemographic characteristics* | | | | |
|  | Age | Information received from the National Population Register | | continuous variable |
|  | Sex | Information received from the National Population Register | | 0= female  1= male |
|  | Educational level | “How many years altogether have you attended school or studied full time? Including primary and comprehensive school.” | Open question | 1= low  2= median  3= high  adjusted for age and sex |
| *Variables related to area of residence* | | | | |
|  | Degree of urbanisation | Information received from the National Population Register | | 1= urban  0= non-urban (semi-urban and rural) |
|  | Distance to local health or social services | “Have the following factors interfered with you receiving  1) treatment and  2) social services in the past 12 months: the place of care/service unit was hard to reach?” | a) always  b) most of the time  c) sometimes  d) never | 0= inconvenient (a–b to either of the questions)  1= convenient (c–d in both questions) |
| *Physical, cognitive, psychological, and social functioning* | | | | |
|  | Functional disability  *Global Activity Limitation Indicator (GALI)* | 1) “Are you limited because of a health problem in activities people usually do?”  2) “Have you been limited for at least the past 6 months?” | 1 a) severely limited  1 b) limited but not severely  1 c) not limited at all  2 a) yes  2 b) no | Only 2 a included  0= severe (1 a)  1= mild or non  (1 b–c) |
|  | Impaired vision | “Can you usually perform the following actions: read ordinary newspaper print (with or  without spectacles)?” | a) yes, with no problem  b) yes, with some difficulties  c) yes, but with great difficulty  d) no, I cannot | 0= yes (c–d)  1= no (a–b) |
|  | The ability to learn new things | “How easily do you learn new things?” | a) very well  b) well  c) adequately  d) poorly  e) very poorly | 0= poor or average  (c–d)  1= good (a–b) |
|  |  |  |  | (continuing) |

| **Variable** | | **Item in questionnaire** | **Response options** | **Coding in the analyses** |
| --- | --- | --- | --- | --- |
| Psychological distress  *Mental Health Inventory (MHI-5)* | | “Over the past 4 weeks, for how much of the time have you felt:  1) very nervous,  2) in such a low mood that nothing could cheer you up,  3) calm and peaceful,  4) downhearted and sad, and  5) happy.” | 1) all the time  2) most of the time  3) a good bit of the time  4) some of the time  5) a little of the time  6) not at all | Items 3 and 5 reverse coded, raw scores converted to a scale from 0 to 100.  0= no (> 53)  1= yes (≤ 52) |
| Living alone | | “Do you live alone?” | a) yes  b) no | 0= yes (a)  1= no (b) |
| *The use of the Internet* | | | | |
|  | Access to the Internet | “Do you have access to the Internet at home, library, or some other place?” | a) no  b) yes | 0= no (a)  1= yes (b) |
|  | Independent use of the Internet | “Do you use the internet for the following:  1) e-services (e.g. My Kanta Pages, MyTax, the Social Insurance Institution of Finland [Kela]),  2) finding information?” | a) I use it independently  b) I use it with another person’s help or someone else uses it on my behalf  c) I don’t use it | 0= no (b–c)  1= yes (a to either of the questions) |
|  | Information security concerns | “How do you feel about the following claims about electronic services: I am concerned about information security when it comes to my personal details?” | a) completely agree  b) somewhat agree  c) neither agree nor  disagree  d) somewhat disagree  e) completely disagree | 0= no (c–e)  1= yes (a–b) |
